# Technical challenge of EUS-guided pancreatic duct drainage with gastroscopy for pediatric pancreaticojejunostomy stricture

**DOI:** 10.1093/gastro/goae109

**Published:** 2025-01-11

**Authors:** Longwei Fang, Zhen Liang, Kun Lian, Senlin Hou, Lichao Zhang

**Affiliations:** Department of General Surgery, The Second Hospital of Hebei Medical University, Shijiazhuang, Hebei, P. R. China; Department of General Surgery, The Second Hospital of Hebei Medical University, Shijiazhuang, Hebei, P. R. China; Department of Biliary and Pancreatic Endoscopic Surgery, The Second Hospital of Hebei Medical University, Shijiazhuang, Hebei, P. R. China; Department of Biliary and Pancreatic Endoscopic Surgery, The Second Hospital of Hebei Medical University, Shijiazhuang, Hebei, P. R. China; Department of Biliary and Pancreatic Endoscopic Surgery, The Second Hospital of Hebei Medical University, Shijiazhuang, Hebei, P. R. China

## Introduction

Surgical resection remains the primary approach for treating childhood tumors to enhance long-term survival. However, postoperative complications pose significant challenges for pediatricians, as they must address both the physical and emotional well-being of young patients, whose developmental stages make them more sensitive. As a result, less invasive procedures are being increasingly favored. Pediatric pancreatic head tumors are exceedingly rare, and managing the postoperative complications associated with such cases is even more unusual. Here, we presented a case involving a pediatric patient with postoperative pancreaticojejunostomy stricture (PJS) following tumor resection, successfully treated with a combination of endoscopic ultrasound (EUS)-guided pancreatic duct drainage and gastroscopy.

## Case report

**Figure 1.**
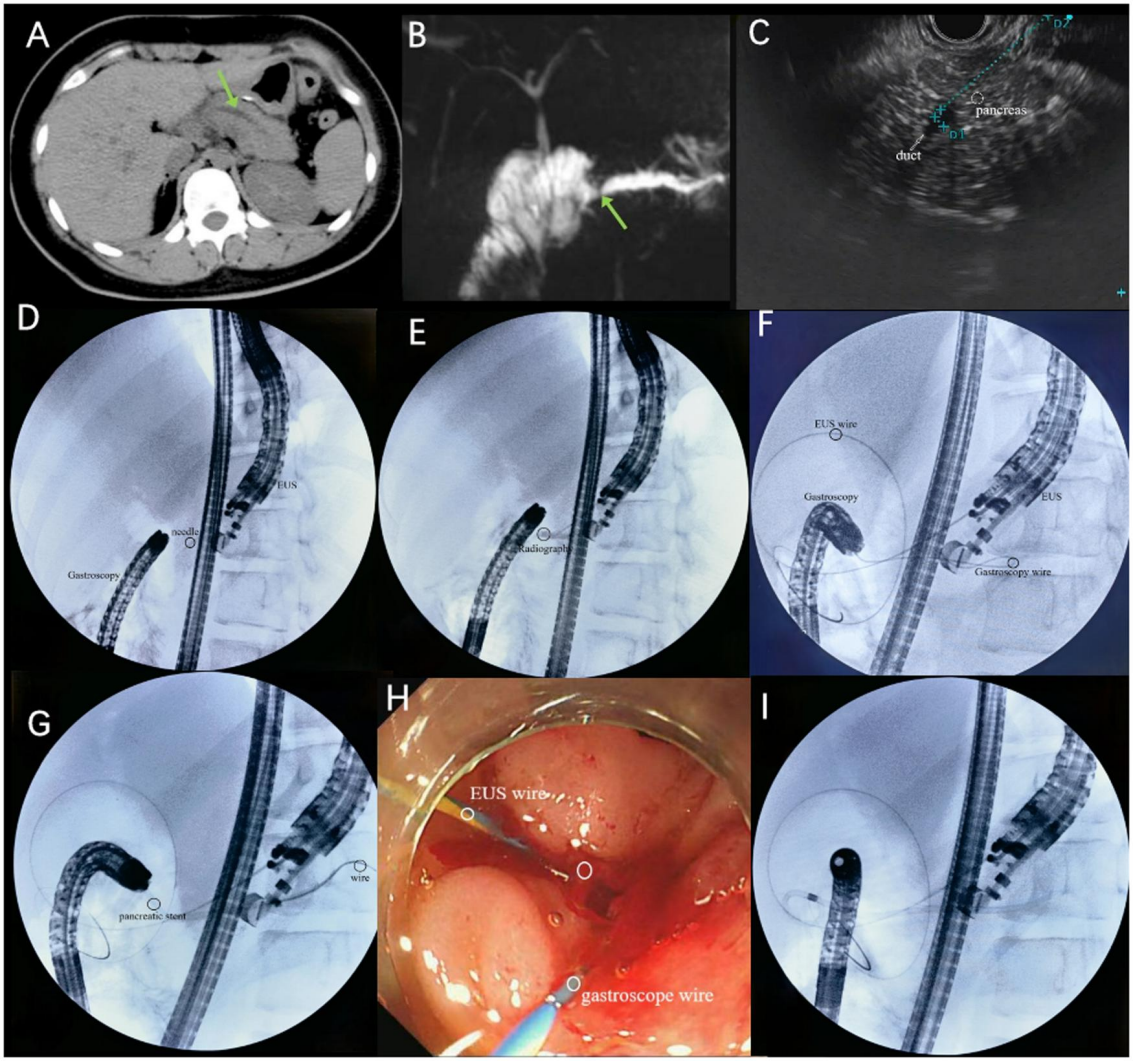
EUS-guided pancreatic duct drainage with gastroscopy for pediatric pancreaticojejunostomy stricture. (**A**) CT shows residual pancreatic tissue with dilated pancreatic ducts. (**B**) Stricture at the pancreaticojejunal junction is shown in magnetic resonance cholangiopancreatography. (**C**) EUS measured the diameter of the pancreatic duct and the distance between the pancreatic duct and the puncture needle. (**D**) Puncturing the pancreatic duct under EUS (black circle). (**E**) The pancreatic duct could be seen when the iohexol was placed along the puncture needle after EUS-PD. (**F**) A guide wire is anterograde placement by EUS-PD and then into the intestinal lumen, the other guide wire is retrograde placement by gastroscopy (black circles shows wires). (**G**) Placement of pancreatic stents along the gastroscopy wire. (**H**) EUS-PD anterograde guide wire and gastroscopy retrograde guide wire (white circle). (**I**) Complete stent placement.

A 12-year-old girl was admitted with a 6-month history of epigastric pain and vomiting, worsening over the past 2 months. One year prior, she had undergone pancreaticoduodenectomy (PD) for a solid pseudopapillary tumor in the pancreatic head. Six months post-operation, she began experiencing recurrent abdominal pain and vomiting. Physical examination revealed tenderness below the xiphoid process, and laboratory tests showed an elevated amylase level of 244.3 U/L. Computed tomography (CT) and magnetic resonance cholangiopancreatography imaging confirmed pancreatic duct dilatation (0.4 cm) in the body and tail (Figure 1A and B), with appendicitis, cholecystitis, and chronic intestinal obstruction ruled out. Based on imaging, clinical history, and elevated amylase, an anastomotic stenosis was suspected, causing increased pancreatic pressure, chronic abdominal pain, and vomiting. The patient was initially scheduled for retrograde pancreatic duct drainage to address the PJS. However, when X-ray imaging revealed that the gastroscope with a transparent cap could not pass the narrow anastomosis, we opted for EUS-guided drainage combined with gastroscopy. EUS revealed pancreatic duct dilatation (Figure 1C), and a 19G needle was used to penetrate the duct (Figure 1D). Following iohexol injection for duct visualization (Figure 1E), the needle guided by a wire was advanced into the jejunum (Figure 1F). X-ray fluoroscopy and gastroscopy then confirmed guide wire positioning at the anastomosis, allowing successful intubation along the guide wire (Figure 1F). Finally, a 7-cm, 7 Fr pancreatic duct stent was placed in the duct via retrograde radiography (Figure 1G and I). Postoperatively, the patient’s recovery was favorable with a stable mood and no significant discomfort. Hemoglobin remained normal, and amylase was measured at 228 U/L. Mild abdominal pain on Day 1 resolved significantly compared to preoperative levels, with amylase normalizing and abdominal pain fully resolving by Day 3. She was discharged 17 days after the operation. At 8 months post-operation, CT imaging showed that the stent had dislodged, but significant improvement in duct dilation remained. At 9 months, the patient reported no recurrent abdominal pain.

## Discussion

PD is rare in pediatric cases, with PJS being even rarer. The choice between endoscopic and surgical intervention for PJS remains debated [1]. Treatment now considers both the physical and psychological impact on patients, especially on children. In this case, the prospect of a second surgery caused significant anxiety in the patient, manifesting as insomnia, irritability, panic, and tearfulness. These factors led us to prioritize endoscopic treatment, given both the psychological considerations and persistent abdominal pain. ERCP is the standard first-line endoscopic treatment for PJS [2]. However, when ERCP fails, EUS-guided PD provides an alternative by creating a transmural channel between the duct and gastrointestinal tract, achieving an efficacy rate of 81% [3, 4]. Fully covered self-expandable metal stents (FCSEMS) are preferred over plastic stents for their greater stability, reduced hospital stays, and lower rates of intervention [5]. In our case, the plastic stent dislodged during follow-up, suggesting that FCSEMS could be a better option in similar cases to prevent stent migration. Additionally, percutaneous pancreatic stent placement is emerging as a feasible supplementary approach [6].

This approach presents several technical challenges. The smaller esophagus in children raises the risk of perforation when inserting a dual endoscope. During scanning, it is crucial to monitor the pancreatic duct and anastomosis carefully to ensure the guide wire can pass through and enter the intestinal cavity. The procedure allows only one puncture, as removing and reinserting the needle could introduce gas into the duct, complicating the operation. After the puncture, the EUS wire must be directed precisely into the intestinal cavity, and careful, gentle retraction of the guide wire is essential to avoid damaging the puncture site and anastomosis. In this case, the EUS was not withdrawn after advancing the guide wire to the jejunum to minimize perforation risk and prevent guide wire displacement. While EUS represents a significant advancement, it does not fully replace traditional surgery. Studies have confirmed that both longitudinal pancreaticojejunostomy and laparoscopic revision of PJS are effective, with no recurrence noted in follow-ups of up to 3 years [2, 7, 8]. However, these findings are limited by small sample sizes, underscoring the need for larger, multicenter studies to establish definitive treatment protocols.

At present, the optimal approach for treating PJS remains controversial; however, endoscopic therapy offers significant psychological benefits by reducing patient stress. Based on our case reports and related literature, we favor endoscopic treatment. When placing stents, careful consideration should be given to the patient’s age and the degree of pancreatic duct dilation to ensure optimal outcomes.

## Authors’ Contributions

L.F. wrote the manuscript, collected information, and put forward the significance of the article; Z.L. and K.L. collected information, assisted in writing; S.H. instructed the treatment and provided the medical scheme; L.Z. revised the paper for important intellectual content. All authors read and approved the final manuscript.
